# Experimental Autoimmune Encephalomyelitis (EAE)-Induced Elevated Expression of the E1 Isoform of Methyl CpG Binding Protein 2 (MeCP2E1): Implications in Multiple Sclerosis (MS)-Induced Neurological Disability and Associated Myelin Damage

**DOI:** 10.3390/ijms18061254

**Published:** 2017-06-12

**Authors:** Tina Khorshid Ahmad, Ting Zhou, Khaled AlTaweel, Claudia Cortes, Ryan Lillico, Ted Martin Lakowski, Kiana Gozda, Michael Peter Namaka

**Affiliations:** 1 College of Pharmacy, Faculty of Health Sciences, University of Manitoba, Manitoba, Winnipeg, MB R3E 0T5, Canada; khorshit@myumanitoba.ca (T.K.A.); zhout346@myumanitoba.ca (T.Z.); khaledta72@gmail.com (K.A.); claudiacortesp@gmail.com (C.C.); umlillic@myumanitoba.ca (R.L.); Ted.Lakowski@umanitoba.ca (T.M.L.); gozdak@myumanitoba.ca (K.G.); 2 College of Pharmacy, Third Military Medical University, Chongqing 400038, China; 3 Department of Medical Rehabilitation, College of Medicine, Faculty of Health Sciences, Winnipeg, MB R3E 0T6, Canada; 4 Department of Internal Medicine, College of Medicine, Faculty of Health Sciences, University of Manitoba, Winnipeg, MB R3A 1R9, Canada

**Keywords:** multiple sclerosis (MS), experimental autoimmune encephalomyelitis (EAE), methyl CpG binding protein 2 (MeCP2), brain-derived neurotrophic factor (BDNF), neurological disability scoring (NDS), myelin repair

## Abstract

Multiple sclerosis (MS) is a chronic neurological disease characterized by the destruction of central nervous system (CNS) myelin. At present, there is no cure for MS due to the inability to repair damaged myelin. Although the neurotrophin brain derived neurotrophic factor (BDNF) has a beneficial role in myelin repair, these effects may be hampered by the over-expression of a transcriptional repressor isoform of methyl CpG binding protein 2 (MeCP2) called MeCP2E1. We hypothesize that following experimental autoimmune encephalomyelitis (EAE)-induced myelin damage, the immune system induction of the pathogenic MeCP2E1 isoform hampers the myelin repair process by repressing BDNF expression. Using an EAE model of MS, we identify the temporal gene and protein expression changes of MeCP2E1, MeCP2E2 and BDNF. The expression changes of these key biological targets were then correlated with the temporal changes in neurological disability scores (NDS) over the entire disease course. Our results indicate that MeCP2E1 mRNA levels are elevated in EAE animals relative to naïve control (NC) and active control (AC) animals during all time points of disease progression. Our results suggest that the EAE-induced elevations in MeCP2E1 expression contribute to the repressed BDNF production in the spinal cord (SC). The sub-optimal levels of BDNF result in sustained NDS and associated myelin damage throughout the entire disease course. Conversely, we observed no significant differences in the expression patterns displayed for the MeCP2E2 isoform amongst our experimental groups. However, our results demonstrate that baseline protein expression ratios between the MeCP2E1 versus MeCP2E2 isoforms in the SC are higher than those identified within the dorsal root ganglia (DRG). Thus, the DRG represents a more conducive environment than that of the SC for BDNF production and transport to the CNS to assist in myelin repair. Henceforth, the sub-optimal BDNF levels we report in the SC may arise from the elevated MeCP2E1 vs. MeCP2E2 ratio in the SC that creates a more hostile environment thereby preventing local BDNF production. At the level of transcript, we demonstrate that EAE-induces the pathological enhanced expression of MeCP2E1 that contributes to enhanced NDS during the entire disease course. Thus, the pathological induction of the MeCP2E1 isoform contributes to the disruption of the normal homeostatic signaling equilibrium network that exists between cytokines, neurotrophins and chemokines that regulate the myelin repair process by repressing BDNF. Our research suggests that the elevated ratio of MeCP2E1 relative to MeCP2E2 may be a useful diagnostic marker that clinicians can utilize to determine the degree of neurological disability with associated myelin damage. The elevated MeCP2E1 vs. MeCP2E2 ratios (E1/E2) in the SC prevent BDNF from reaching optimal levels required for myelin repair. Thus, the lower E1/E2 ratios in the DRG, allow the DRG to serve as a weak secondary compensatory mechanism for enhanced production and delivery of BDNF to the SC to try to assist in myelin repair.

## 1. Introduction

Multiple sclerosis (MS) is a chronic progressive neurological disease of central nervous system (CNS) characterized by destruction of myelin [1,2]. It is considered a biphasic autoimmune disease with an acute inflammatory phase followed by a chronic degenerative, demyelinating phase [3]. Myelin is the insulating coating that surrounds nerve axons. It is essential for propagation of nerve impulses to effector target cells. Hence, patients diagnosed with MS have regionalized areas along axons where electrical impulse transmission is compromised due to damaged segments of myelin. Thus, MS patients suffer a variety of neurological disabilities that negatively impact their quality of life [1,4].

Using an animal model of MS, we have published several publications that identify the importance of the anatomical connection between the dorsal root ganglia (DRG) and spinal cord (SC) [5,6,7] in regard to myelin repair. In addition, several of our publications have identified a common interactive signaling network among cytokines, chemokines and neurotrophins that regulate the myelin repair process [6,7,8,9]. We have identified several key biological targets in this pathway [5,7,8,9,10,11], However, we have now expanded this list of biological targets to include the upstream transcriptional repressor of BDNF known as methyl CpG binding protein 2 (MeCP2) [11,12,13]. 

BDNF has a well-established role in myelin repair [12,14,15,16]. It has been shown to be involved in proliferation, differentiation and migration of oligodendrocyte progenitor cells (OPCs) [17,18]. Furthermore, BDNF also has a direct effect on myelination via its effect on oligodendrocytes (OGs) [19,20,21]. In addition, BDNF has been shown to regulate the distribution pattern of myelin proteins that govern in the structural integrity of myelin [22,23,24]. Other researchers in this area have also confirmed the beneficial effects of BDNF in re-myelination and/or myelin repair. For example, several studies have shown that fingolimod [25], the first approved oral drug for MS, glatiramer acetate [26,27,28] and laquinimod [29,30] exert their beneficial effect in treating relapsing remitting MS (RRMS) by increasing BDNF levels. In addition to these findings, we have also published evidence to support the role of BDNF via tropomyosin-related kinase receptors (TrkB) in SC myelin repair [11].

**Figure 1:**
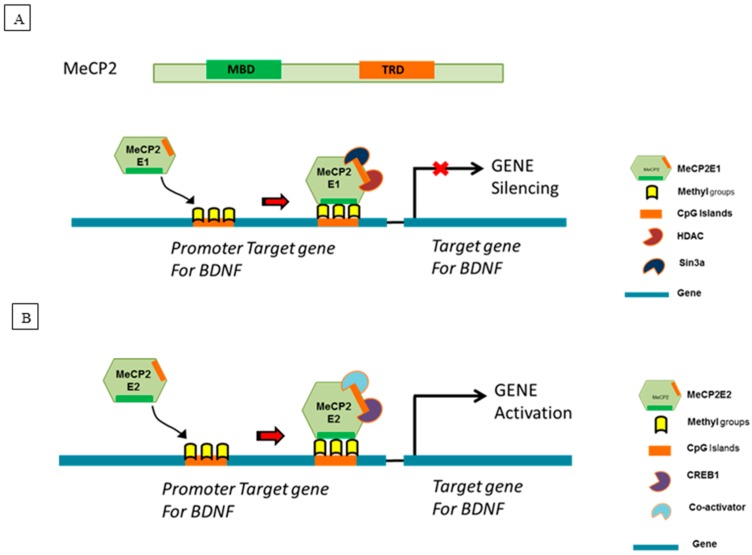
Proposed molecular mechanism of action of MeCP2E1 and MeCP2E2. *BDNF* gene is very complex. Aid et al. described 10 exons in human and rodent *BDNF* gene [44]. MeCP2 has been shown to bind to promoter region of exon III in rats [35] and exon IV in mice and humans. (**A**) Proposed MeCP2E1 mechanism of gene repression: MeCP2E1 binds to methylated CpG dinucleotide in CpG islands of promoter region for *BDNF* gene with its methyl binding domain (MBD) and recruits a transcriptional co-repressor complex containing mSin3A and histone deacetylase 1 (HDAC1) and 2 (HDAC2) with its transcriptional repressor domain (TRD) to repress the expression of BDNF during an MS attack leading to MS induced myelin damage; (**B**) Proposed MeCP2E2 mechanism of gene activation: MeCP2E2 binds to methylated CpG dinucleotide in CpG islands of promoter region for *BDNF* gene and recruits a transcriptional activator complex containing cAMP response element-binding protein (CREB) and co-activators [45]. This leads to activation of *BDNF* gene during an MS attack in order to help in re-myelination and/or myelin repair.

Since its identification in 1998 [31], MeCP2 has received a wide range of attention as a genome-wide epigenetic factor [32]. In fact, MeCP2 was the first member of methyl binding protein that was able to bind a single methyl CpG pair [33] by its methyl binding domain (MBD) [34]. BDNF is one of several prominent targets of MeCP2 [35,36]. MeCP2 was first identified to be a transcriptional repressor of BDNF [35,37] exerting its effect by directly forming a repressor complex with its transcriptional repressor domain (TRD) and histone deacetylase (HDAC) Sin3A complex [38,39] (Figure 1A). MeCP2 can also indirectly regulate BDNF gene expression by affecting the expression of other BDNF transcriptional repressors like RE1 silencing transcription factor (REST) and CoREST [40]. Interestingly, studies conducted in MeCP2 null mice [36,41,42] and human tissues [43] show decreased gene and protein expression of BDNF. Thus, these reported findings in MeCP2 null mice suggest that other factors must be involved in the regulation of BDNF expression besides MeCP2. Irrespective, MeCP2’s ability to directly and/or indirectly effect BDNF expression warrants its investigation as a primary biological target by which targeted interventional strategies could be developed to promote myelin repair.

MeCP2 exists in two different biologically active isoforms, MeCP2E1 and MeCP2E2 [46,47]. Both isoforms have been shown to have differential biological effects on neuronal survival [48] and embryonic development [49]. Based on our current investigative research in this area [50], we believe these two isoforms may have distinctly different biological roles in regard to the re-myelination and/or the myelin repair process that may be linked to their ability to differentially regulate the expression of BDNF [13,35,37,51,52,53]. Thus, the MeCP2E1 isoform may be pathogenically activated by an immune system mediated insult such as in MS. The resultant disease-induced elevation of MeCP2E1 represses BDNF production that alters the homeostatic molecular signaling between cytokines, chemokines and neurotrophins resulting in myelin damage [50]. For example, studies have confirmed repression of BDNF by MeCP2E1 in an inflammatory stimulus induced pain model [54,55]. Specifically, this study showed increased expression of the MeCP2E1 isoform in an inflammatory pain model that utilized complete Freund’s adjuvant (CFA) injection as the nociceptive inflammatory pain stimulus [54]. This increase was also correlated with increase HDAC1 and HDAC2 levels both of which are part of MeCP2 repressor complex [54]. Recent research suggests that the MS induced pathological production of MeCP2 may be the result of epigenetic effects. For example, recent epigenetic studies involving changes in DNA methylation and histone acetylation have also linked MeCP2 to MS [56,57]. Conversely, the MeCP2E2 isoform may be involved in myelin repair by promoting BDNF production by activating the BDNF gene (Figure 1B). However, detailed research in this newly evolving area is lacking, thus additional studies are required before we can draw any definitive conclusions in this regard. 

Interestingly, MeCP2 has also been shown to be involved in regulation of myelin gene expression thereby governing the structural integrity of myelin [51,58]. A recent study in MeCP2 knock down cultured oligodendrocytes identified its effects on the transcriptional induction of myelin proteins [58]. These studies suggest that MeCP2 may be critical component that regulates the structural integrity of myelin by altering the ratios of the myelin structural proteins that comprise myelin. 

**Figure 2:**
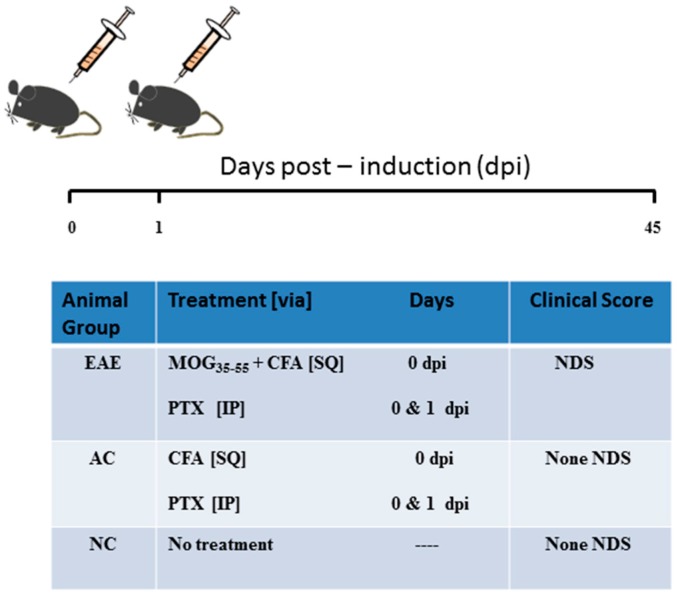
Experimental autoimmune encephalomyelitis (EAE) induction by myelin oligodendrocyte glycoprotein (MOG). Our EAE animals were immunized with 200 µg MOG_35–55_ in 200 µL of complete Freund’s adjuvant (CFA) subcutaneously (SQ) on Day 0. Active control (AC) animals only received the CFA adjuvant. Both EAE and AC animals received intraperitoneal (IP) injections of pertussis toxin (PTX) on Days 0 and 1. Naïve control (NC) did not receive any treatment. EAE = experimental autoimmune encephalomyelitis, AC = active control, NC = naïve control, MOG = myelin oligodendrocyte glycoprotein, CFA = complete Freund’s adjuvant, PTX = pertussis toxin, NDS = neurological disability scoring, dpi = days post induction, SQ = subcutaneous, IP = intraperitoneal.

To the best of our knowledge, there are no other studies that have evaluated the differential expression of the two biologically active but different isoforms of MeCP2 in experimental autoimmune encephalomyelitis (EAE) animal model of MS (Figure 2). Given the wealth of literature linked MeCP2 to myelin damage in neurological conditions such as Rett Syndrome [53,59,60], it became apparent the importance of conducting similar research studies in other white matter disorders such as MS. Thus, this is the first study to demonstrate the pathological effects of the MeCP2E1 in regard to its ability to suppress the expression of BDNF resulting in severe neurological disability scoring (NDS) and associated myelin damage. Specifically, our current research study examines the temporal gene expression of MeCP2E1, MeCP2E2 and BDNF in SC tissue obtained from a myelin oligodendrocyte glycoprotein (MOG)-induced model of EAE. In addition, we correlated these temporal changes in gene expression with the temporal changes in NDS during the acute and chronic phases of disease progression.

Our current study identifies the importance of the pathological induction of MeCP2E1 in an EAE animal model of MS. Our results suggests that EAE-induced expression of MeCP2E1 (transcriptional repressor of BDNF) contributes to the sub-optimal levels of BDNF resulting in sustained elevated NDS with associated myelin damage. Our research also suggests the pathogenic involvement of immune system mediated induction of MeCP2E1 repression of BDNF. Based on our previously published findings in this area, we believe that this EAE-induced MeCP2E1 expression contributes in part to the disruption of the homeostatic signaling balance between cytokines, chemokines and neurotrophins resulting in enhanced neurological disability with associated myelin damage.

## 2. Results

### 2.1. Neurological Disability Scoring (NDS)

**Figure 3:**
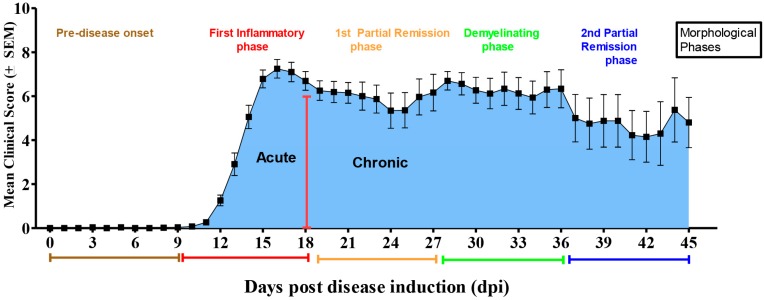
Clinical course of EAE-induced neurological disability in MOG-induced EAE mice. Preliminary results of mean neurological disability scores recorded in MOG-induced EAE mice at different time points of disease progression. For example, Days 3, 6, and 9 (pre-disease onset), Days 12, 15, and 18 (first inflammatory phase prior to de-myelination), Days 21, 24 and 27 (during first partial incomplete remission), Days 30, 33, and 36 (demyelinating phase), and Days 39, 42 and 45 (during the second period of partial incomplete remission: re-myelination phase). Mean global neurological disability scores were obtained following assessment of all six specific clinical domains. Neurological disability scores (NDS) range from a score of 0 (no disability) to 15 (maximal disability). Our preliminary results indicate that MOG-induced EAE mice exhibit clinical signs of the disease activity at ~11 days after the initial immunization (disease onset phase) that peaked at approximately Days 17 (pre-demyelinating disease phase) and 27 (de-myelinating disease phase). We also observed the remission (re-myelinating phase) at Day 21.

All animals in the EAE groups underwent NDS. All animals in the NC and AC experimental groups displayed a normal NDS of zero. Based on our NDS analysis, we observed an acute disease phase that became apparent during Days 9–18 post-induction. We also observed a chronic disease phase that started between Days 18 and 45 post-induction. Our identified phases of disease progression are consistent with other researchers that have used the same MOG model of EAE [2]. Specifically, we identified that during the acute phase animals begin to develop mild clinical symptoms by Day 10 post-induction (tail weakness or paralysis) [61] (Figure 3). By Days 12–13 post-induction, all animals experience a full range of clinical neurological deficits such as tail and forelimb weakness, loss of bladder control and hind-limb paralysis. As the disease characteristically progresses, the mice enter into a slight remission and regain motor function by Day 18 post-induction. The chronic disease phase is followed by a second peak of neurological disability (associated with de-myelination) at 28–30 days post-induction with another slight remission phase identified from Days 39–45 post-induction. The control groups (NC and AC) did not show any clinical signs of disability (data not shown). 

### 2.2. mRNA Expression of MeCP2E1 and MeCP2E2 Isoforms in the SC

**Figure 4:**
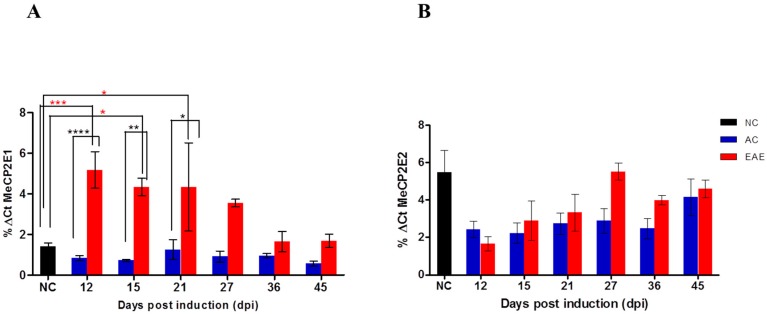
RNA expression of MeCP2 isoforms in the Spinal Cord (SC). (**A**) Quantitative real time polymerase chain reaction (RT-PCR) shows MeCP2E1 mRNA gene expression in SC at different times in the disease progression. %Δ*C*_t_ analysis of real time RT-PCR of MeCP2E1-EAE animals show a significant increase of MeCP2E1 expression in SC over NC animal groups at 12, 15 and 21 dpi. EAE animals show a significant increase of MeCP2E1 expression in SC over AC at 12, 15 and 21 dpi. In comparison, NC and AC do not show significant difference at all different time points; (**B**) Quantitative RT-PCR shows MeCP2E2 mRNA gene expression in SC at different times in the disease progression. %Δ*C*_t_ analysis of real time RT-PCR of MeCP2E2 expression in SC do not shows significant difference in mRNA expression between the NC control animal and EAE group. (**** *p* < 0.0001; *** *p* < 0.001; ** *p* < 0.01; * *p* < 0.05). (Two-way ANOVA followed by Bonferroni’s post *hoc* test). Values are mean ± SEM.

In order to determine which MeCP2 isoform contributes to the regulation of BDNF during EAE disease course, we quantified the changes in MeCP2E1 and MeCP2E2 gene expression in the SC. Quantitative real time polymerase chain reaction (qRT-PCR) analysis was conducted on SC tissue isolated from the three experimental groups (EAE, NC and ACs), at the pre-determined experimental time points (Figure 4A). The MeCP2E1 mRNA expression was assessed in parallel with that of the housekeeping gene (GAPDH). Following Δ*C*_t_ analysis, EAE animals (red bar) show a significant increase of MeCP2E1 expression in SC over NC (black bar) and AC (blue bars) animal groups at 12, 15 (acute disease phase) and 21 dpi (chronic disease phase). However, NC and AC do not show significant difference at all different time points. We used the same method to evaluate gene expression for MeCP2E2 in SC on our three experimental groups (EAE, NC and ACs), at the pre-determined experimental time points (Figure 4B). However, we observed no significant differences in expression among our groups.

### 2.3. BDNF Gene and Protein Expression in SC

**Figure 5:**
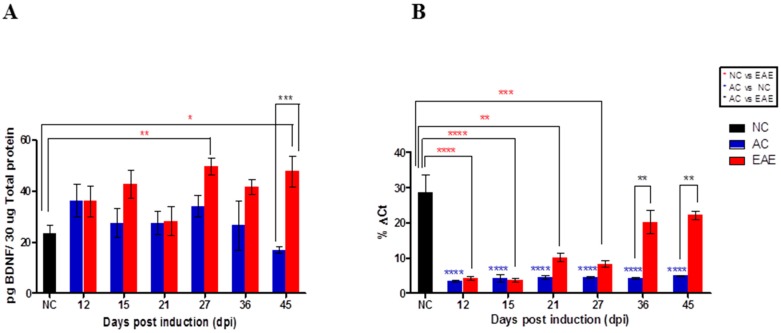
Transcript and protein expression of BDNF in the spinal cord (SC). (**A**) ELISA quantification of BDNF expression in the SC. BDNF expression in the SC was quantified using ELISA. There is significant differences between NC and EAE at Day 27 (** *p* < 0.01) and 45 (* *p* < 0.05) and between AC and EAE (*** *p* < 0.001) at Day 45. (Two-way ANOVA followed by Bonferroni’s post hoc test). Values are mean ± SEM; (**B**) RT-PCR quantification of BDNF mRNA expression in the SC. %Δ*C*t analysis of real time RT-PCR of BDNF expression in SC shows significant differences in mRNA expression between the NC animals (black bars) and EAE (red bars) at 12, 15, 21 and 27 dpi. EAE group show significant increase compared to AC animal (blue bars) at 36 and 45 dpi. There are significant differences between NC and AC at all-time points. (**** *p* < 0.0001; *** *p* < 0.001; ** *p* < 0.01; * *p* < 0.05). (Two-way ANOVA followed by Bonferroni’s post hoc test). Values are mean ± SEM.

BDNF is involved in re-myelination and is regulated by MeCP2 [35,36]. Therefore, we conducted gene and protein quantification of BDNF in SC tissue obtained from NC, AC and EAE animals to determine if regulation of BDNF expression is isoform specific (Figure 5). ELISA was employed to quantify differential protein expression for BDNF. For each sample, results are given as pg BDNF per 30 µg total protein. We identify significant differences between NC, and EAE (** *p* < 0.01) at Day 27 post-induction that corresponds to the second relapse phase of the disease and also at Day 45 (* *p* < 0.05) that corresponds to second partial remission phase of the disease (Figure 3). In addition, there are significant differences between EAE and AC (*** *p* < 0.001) at Day 45 post-induction (Figure 5A). For comparison we measured BDNF gene expression in SC, via qRT-PCR. Significant differences exist between the NC animals (black bars) and EAE (red bars) at Days 12 (**** *p* < 0.0001), 15 (**** *p* < 0.0001), 21 (** *p* < 0.01) and 27 (*** *p* < 0.001) post-induction. There are also significant differences between EAE and AC (blue bars) at Day 36 and 45 post-induction (** *p* < 0.01). Finally, NC and AC showed significant differences (**** *p* < 0.0001) at all-time points (Figure 5B).

### 2.4. MeCP2E1 and MeCP2E2 Protein Expression in SC during Acute and Chronic Disease Phase of EAE

**Figure 6:**
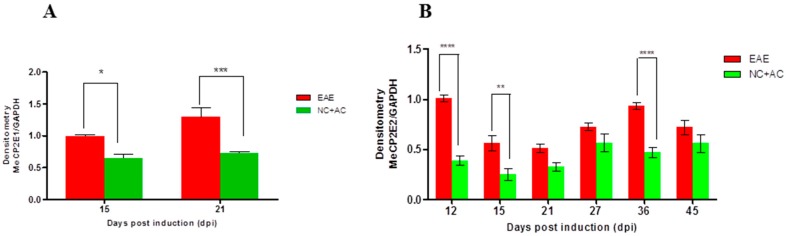
MeCP2E1 and MeCP2E2 protein expression in SC during acute and chronic disease phase of EAE. (**A**) Western blot quantification of MeCP2E1 expression in SC. Results are shown as the ratio of MeCP2E1/GAPDH. EAE animals show a significant increase of MeCP2-E1 expression in SC over NC+ AC animal groups at 15 and 21 dpi. (*** *p* < 0.001; * *p* < 0.05). (Two-way ANOVA followed by Bonferroni’s post hoc test). Values are mean + SEM; (**B**) Western blot quantification of MeCP2E2 expression in SC. Results are shown as the ratio of MeCP2E2/GAPDH EAE animals show a significant increase of MeCP2-E2 expression in SC over NC+ AC animal groups at 12, 15 and 36 dpi. (**** *p* < 0.0001; ** *p* < 0.01). (Two-way ANOVA followed by Bonferroni’s post hoc test). Values are mean ± SEM.

**Figure 7:**
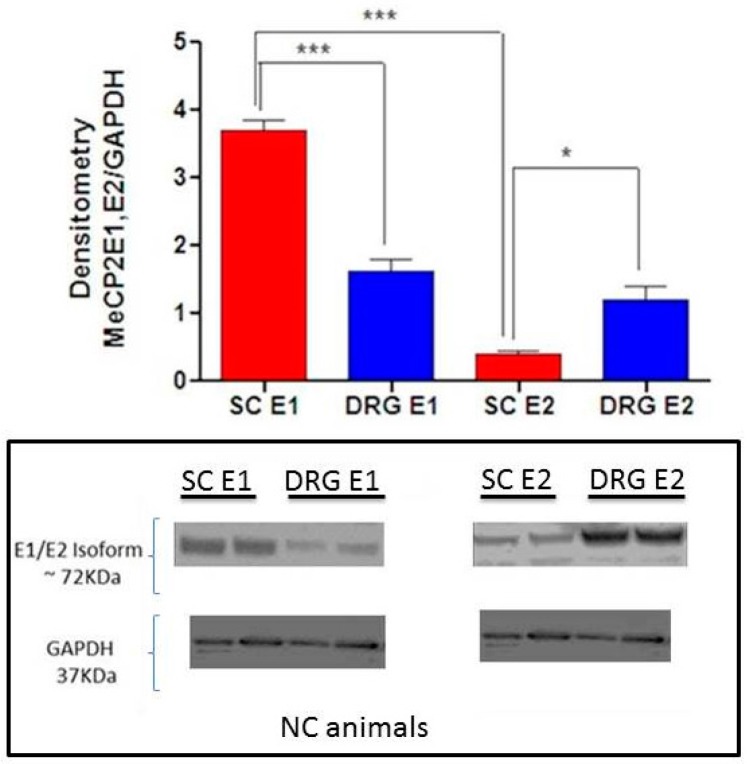
Baseline MeCP2E1 and MeCP2E2 expression in DRG and SC of NC animals. Results are shown as the ratio of MeCP2E1/GAPDH and MeCP2E2/GAPDH. There are significant differences between MeCP2E1 (*** *p* < 0.001) and MeCP2E2 (* *p* < 0.05) expression level in DRG and SC. Relative expression of MeCP2E1/ MeCP2E2 is 9.25 in SC, whereas it is 1.36 in DRG. SC E1 = protein expression of MeCP2E1 in spinal cord, DRG E1 = protein expression of MeCP2E1 in dorsal root ganglia, SC E2 = protein expression of MeCP2E2 in spinal cord, DRG E2 = protein expression of MeCP2E2 in DRG.

To compare MeCP2E1 and MeCP2E2 protein expression during the acute and chronic phase in SC, we conducted Western blot. Quantification of MeCP2E1 protein expression shows a significant increase for EAE over NC+ AC animal groups at 15 (* *p* < 0.05) and 21 dpi (*** *p* < 0.001) (Figure 6A). Furthermore, EAE animals show a significant increase of MeCP2E2 expression in SC over NC + AC animal groups at 12 (**** *p* < 0.0001), 15 (** *p* < 0.01) and 36 dpi (**** *p* < 0.0001) (Figure 7B). 

### 2.5. Baseline Protein Expression of MeCP2E1 and MeCP2E2 Isoforms in Spinal Cord (SC) and Dorsal Root Ganglia (DRG) in Naïve Control (NC) Animals

In order to compare protein expression of MeCP2E1 and MECP2E2 isoforms in the DRG and SC, we conducted WB analysis. Quantification of MeCP2E1 and MeCP2E2 protein by WB densitometry in the SC and DRG indicates significant differences between MeCP2E1 (*** *p* < 0.001) and MeCP2E2 (* *p* < 0.05) expression level (Figure 7). Relative expression ratio of MeCP2E1/MeCP2E2 is 9.25 in SC, versus a ratio of 1.36 in DRG. 

## 3. Discussion

At present, the exact pathophysiological molecular mechanisms underlying MS-induced neurological deficits due to associated myelin damage is still unknown. However, BDNF has been suggested as one of the lead biological target molecules due to its well established beneficial role in myelin repair [19,20,21,62]. For example, BDNF has been shown to regulate OPCs via molecular signaling through TrkB receptors to induce their proliferation, migration and differentiation [18]. Recent MS research has turned to other white matter neurological disorders such as Rett Syndrome, to provide insight into the additional factors that may regulate the transcriptional expression of BDNF. Based on the research conducted in Rett Syndrome, researchers have only recently identified MeCP2 as an upstream transcriptional repressor of BDNF [35,37,52,53].

In our current study we hypothesized that transcriptional repression of BDNF by the EAE-induced upregulation of MeCP2E1 disrupts homeostatic signaling network equilibrium between inflammatory cytokines, chemokines and neurotrophins resulting in elevated NDS due to associated myelin damage. To address this issue we used a MOG-mouse model of EAE. EAE animals characteristically exhibited two distinct disease phases, acute and chronic. The acute phase includes inflammation, displaying early signs of myelin damage that leads to peak NDS at Day 18 followed by a chronic demyelinating disease phase that encompasses Days 18–45 (Figure 3). The disease pattern displayed in our animal model is consistent with the relapsing remitting models previously described in other studies [63]. 

Based on our current study, we are the first researchers to show the differential expression of the biologically active MeCP2E1 and MeCP2E2 isoforms at the mRNA level in a MOG-induced EAE model of MS. In addition, we are also the first to demonstrate that MeCP2E1 and MeCP2E2 isoforms regulate the temporal changes in BDNF gene and protein expression. Our results demonstrated significant elevations in the mRNA expression levels of the MeCP2E1 isoform (relative to NCs and ACs) during the first inflammatory acute phase of disease prior to de-myelination (EAE 12–18) (Figure 4A). These results correlated with our observed significant decrease in BDNF mRNA expression (Figure 5B) relative to NC’s that were also associated with elevated NDS during this same time period (Figure 3). Thus, our results suggest that the EAE-induced MeCP2E1 expression, contributed to the repressed BDNF expression resulting in sustained elevated NDS known to occur during an EAE-induced attack on CNS myelin. According to our reported data, the progressive decreasing levels of MeCP2E1 isoform from EAE 21–27 (first slight remission phase) appeared to promote the marked increase in BDNF mRNA expression at these later time points. However, since the mRNA levels for BDNF did not rise to significant levels above that of NC’s, we believe that these sub-optimal levels failed to facilitate re-myelin repair resulting in sustained elevations in NDS even in the later stages of disease. (Figure 3, Figure 4A and Figure 5B). Although, our results show that MeCP2E1 isoform mRNA expression continually decreased during the chronic phase of disease progression (EAE 30–36), the lower levels of MeCP2E1 were still higher than those of NC and AC animals. Thus, we believe that the persistent elevated MeCP2E1 levels that even extend out to the later time points of the chronic disease phase (EAE 39–45) are responsible in part for the continued repression of BDNF (Figure 5) that results in the sustained elevations in NDS with associated myelin damage. Hence, the EAE animals never achieve full neurological recovery, as evident by the persistent elevations in NDS that never returns to baseline (Figure 3, Figure 4A and Figure 5A,B). Overall, our findings suggest that the persistent elevated levels of MeCP2E1 in the SC, creates a hostile environment that prevents the localized production of BDNF protein from ever reaching the required optimal physiological levels that are beneficial for myelin repair. Based on the very limited availability of antibodies the protein analysis for the MeCP2E1 and MeCP2E2 isoforms were not conducted. However, recent research involving the use of a C-terminal anti-MeCP2 antibody raised to the C-terminus may prove to be of significant importance to other researchers conducting this type of analysis [47].

During the acute disease phase (EAE 12 and EAE 15), the MeCP2E2 protein expression levels are elevated relative to NC + AC in an attempt to increase BDNF production and facilitate re-myelination and/or myelin repair during the initial EAE insult (Figure 6B). Similarly, during the chronic disease phase, MeCP2E2 protein levels remain elevated with a second significant peak at ~EAE 36, which corresponds to a significant surge of BDNF protein production in an attempt to increase BDNF production and facilitate re-myelination and/or myelin repair. Overall, the elevated protein levels of MeCP2E2 during the entire course of EAE attempt to counteract the damaging BDNF repressive effects of MeCP2E1. However, due to high MeCP2 E1/E2 ratio of 1.6 and 2.6, during the acute and chronic phase (EAE 15 and 21) (Figure 6A), MeCP2E2 protein levels never fully reach the desired levels to accomplish complete BDNF induced re-myelination and/or myelin repair with associated full neurological recovery. Thus, EAE animals are left with residual neurological deficits and at best only achieve partial remissions during the course of the disease.

While the differential expression of two isoforms of MeCP2 in the SC compared to the DRG is of interest (Figure 7), this observation could also be due to differential representation of neuronal vs. non-neuronal nuclei. However, detailed studies focused on identifying the exact cellular sources of the isoforms including manipulation of the expression of the different isoforms in relevant cell types represent an essential research direction for future studies that include SC and DRG tissues. These studies will be instrumental in confirming the importance of the DRG-SC anatomical connection in the myelin repair process. Although our current study did not evaluate the anterograde transport of BDNF from DRG to SC, our previous publication demonstrated BDNF transport from DRG to SC by kinesin protein that support this concept [8]. Thus, the ratio of MeCP2E1 versus MeCPE2E2 in the DRG and SC may be of critical importance as a useful clinical diagnostic test as a measure of the degree of neurological disability and associated myelin damage.

Studies have suggested that MeCP2 induced transcriptional regulation of BDNF is dependent on several factors including extracellular calcium [35], DNA methylation [37] status and phosphorylation of MeCP2 at serine 421 during neuronal depolarization, all leading to MeCP2 release from the promoter and subsequent transcription of BDNF [35,52]. Martinowich et al. showed this is associated with reduced methylation at promoter site [37]. Thus, their research showed that MeCP2 regulation of BDNF gene is context dependent. Furthermore, recent studies confirm the complexity by which the BDNF gene is regulated. For example, researchers have suggested that the regulation of the BDNF gene depends on the specific cell types, brain structure and promoter region [53]. Hence, although our research demonstrates an association between MeCP2E1 in regards to BDNF repression, it is clearly not the only factor involved in the regulation of the BDNF gene. Adding further complexity to this issue, other researchers have shown the transcriptional activation of BDNF by MeCP2 through cAMP response element-binding protein (CREB) activator complex (Figure 1B) [45,64]. However, these types of studies did not differentiate between the two biological isoforms of MeCP2. Our results show that the MeCP2E2 isoform may have a beneficial role that involves the activation of the BDNF gene. Hence, resultant severe neurological disability caused by associated myelin damage occurs from the EAE-induced increase in the MeCP2E1 isoform relative to MeCP2E2. For instance, the increased expression ratio of MeCP2E1 versus MeCP2E2 has also been reported in the brain tissue obtained from a mouse model of Rett syndrome [65]. However, detailed research in the area of differential biological activity between the two isoforms is just starting to evolve. We must also realize that the BDNF gene is very complex, generating multiple transcripts by splicing of 8 exons, each under a different transcriptional promoter [44]. Hence, additional research is required in specific regard to the mRNA levels of all BDNF splice variants. In addition, further research is also required in regard to the transcriptional regulation by MeCP2E1 and MeCP2E2 at the different BDNF promoter regions. These additional types of studies are required to unveil the exact mechanisms by which the BDNF gene is regulated by MeCP2 and other molecules. 

Our research at the transcript level, also aimed to evaluate the role of MeCP2E2 isoform in regulation of BDNF. We observed that during the first inflammatory acute disease phase prior to de-myelination (EAE 12–18), the MeCP2E2 isoform mRNA expression levels are markedly low relative to NCs (Figure 4B). Interestingly, the marked reduced expression levels of MeCP2E2 correspond to the reduced BDNF levels during the acute disease phase (EAE 12–18) (Figure 5). However, as the EAE disease progressions, our results identify that the MeCP2E2 mRNA levels continue to increase during EAE 21–27 (chronic disease phase) peaking at EAE 27 (Figure 4B). We speculate that the gradual increase in the MeCP2E2 isoform during this time period represents a failed attempt to activate the BDNF gene by the MeCP2E2 isoform. We also speculate that the failure of MeCP2E2 to reach significant levels to counteract the pathological MeCP2E1 expression result in the sustained elevated NDS throughout the acute and chronic phases of disease. Our results also identify that the reduced MeCP2E2 expression during the chronic disease phase (EAE 30–36) (Figure 4B) corresponds in part with the reduction in BDNF expression (Figure 5A). However, this is a weak correlation and further research in regard to the effects of MeCP2E2 activation of BDNF are required to conclusively determine this link as cause or effect. Interestingly, our data presented for MeCP2E2 expression in the chronic disease phase (EAE 39–45) (Figure 4B) identify a marked increase in MeCP2E2 mRNA, that correspond more closely as we would have expected with the reported increases in BDNF protein during the same time period (Figure 5A). Irrespective, additional research in this area is required before definitive conclusions can be drawn in regard to MeCP2E2 effects on BDNF expression.

Although MeCP2E1 and MeCP2E2 isoforms have over 95% similar sequence homology, other studies support our current research findings which suggest the isoforms may have differential biological activity [49]. For example, it has recently been reported that mutations in exon 1 [66,67] have been suggested to implicate MeCP2E1 in causing the white matter damage that is known to occur in Rett syndrome. Furthermore, other studies have also confirmed the differential effects on different target gene expression profiles that are regulated by the MeCP2E1 and MeCP2E2 isoforms [68]. For instance, Milacic et al. evaluated different gene expression patterns for MeCP2 isoforms in neuronal cells over-expressing predominantly MeCP2E1 or MeCP2E2 [68]. They found that many genes involved in axon genesis, neuronal differentiation and tyrosine kinase receptor signaling are regulated by MeCP2E1 [68]. In addition, they also found that genes involved in tyrosine kinase signaling and immune response were down regulated by MeCP2E1 [68]. However, the MeCP2E2 isoform mostly regulates those genes involved in chromatin organization and transcriptional regulation [68]. These functional biological differences may be related to differences in N-terminal sequence of two isoforms that causes shorter half-life for MeCP2E2 [69] and different DNA binding specificity. As a result, the functional differences reported for each MeCP2 isoform may also account for the suggested differential role of each isoform in regard to myelin repair. Hence, targeted attenuation of the pathological MeCP2E1 isoform warrants further investigation to determine the merit in designing epigenetic interventional strategies aimed at restoring a normalized balance between MeCP2E1 and MeCP2E2. However, despite our intriguing results, we also realize that the EAE-induced changes identified for MeCP2 and BDNF may be a cause rather than a consequence of the associated myelin damage following an immune system attack on CNS myelin. In addition, we also realize the importance of identifying the specific cellular source of MeCP2 will be instrumental in designing targeted treatment strategies to promote myelin repair. Thus, future studies are required in these areas in order to help us establish a stronger connection for MeCP2 that is causative rather than consequential. 

Research suggests that both mutations and duplication in MeCP2 could lead to neurodevelopmental abnormalities [70,71]. Thus, it is imperative to keep MeCP2 in a narrow range for normal neurological functioning. Our study also provides supportive evidence in regard to the potential importance of the MeCP2E1/MeCP2E2 ratio in EAE model of MS. The present study suggests that the pathological over-expression of MeCP2E1 represses BDNF resulting in a disruption of the homeostatic signaling equilibrium between cytokines, chemokines and neurotrophins resulting in incomplete re-myelination and/or myelin repair with corresponding neurological disability.

Although the tissue heterogeneity could be an alternative explanation for our results, there are a number of feasible explanations for the changes in expression that are observed that should be considered. For example, the mRNA analysis in the study is performed on whole tissue that is undergoing dramatic inflammation and rearrangement during this experiment. It is very possible that the changes in MeCP2 isoform expression or BDNF expression that are observed arise from changes in cellular composition that occur due to the inflammation. For example, the MeCP2E1 isoform is specifically enriched in neurons, so if the tissue assessed by protein analysis is infiltrated with a large amount of immune cells, the relative contribution of MeCP2E1 relative to loading controls would be expected to go down. The cellular source of MeCP2 in the SC has been evaluated in other studies which have identified astroglia and neurons as the key sources of MeCP2 [72]. However, the focus of this manuscript was to present the molecular changes in the biological targets that our result shows were involved in causing neurological disability through the molecular mechanisms that facilitate myelin damage. Thus, we did not conduct detailed immunohistochemistry and/or in situ hybridization to identify the specific cellular sources of the molecular changes at the gene and protein level. However, identifying the specific cellular source(s) of these molecular changes represents the next phase of research that is required to be conducted now that we have identified and confirmed the changes in the biological targets we believed were responsible for promoting myelin damage. Furthermore, although we have provided data that suggest MeCP2E1 is involved in the transcriptional repression of BDNF, further studies involving MeCP2E1 knockouts must be conducted to confirm its regulation of BDNF in an animal model of MS.

## 4. Materials and Methods

### 4.1. Induction of EAE

The MOG mouse model of EAE is the preferred model of MS [2,73,74]. Ten-week-old C57 BL/6 mice were randomly assigned to either: naïve control (NC), active control (AC) and MOG-induced EAE. A total of *n* = 4 mice were used per time point per group. As per our standard in house protocols [6,7,9,11], EAE mice were immunized subcutaneously (SQ) with 200 µg MOGp35–55 in 200 µL of Complete Freund’s adjuvant (CFA) at the lower/upper back at Day 0 (induction kits from Hooke Laboratories (Lawrence, MA 01843, USA) (Cat, EK-2110)). Animals received two intraperitoneal (IP) injections of pertussis toxin (PTX: List Biological Laboratories; #179B) (0.2 µg in 100 µL of PBS at Days 0 and 1) to open up the blood brain barrier and facilitate the entry of pathogenic T cells to the CNS. AC mice received all the same treatment as the EAE mice with the exception of the omission of the MOG antigen. NC animals did not receive any treatment (Figure 2). The CFA emulsion contains killed mycobacterium tuberculosis (MT) H37Ra in incomplete Freund’s adjuvant (FA) to enhance the immune system response to sensitization. The emulsion was administered SQ at two sites in the upper and lower back area. A 0.1 mL SQ dose of the emulsion was injected at each site (0.2 mL/mouse) at Day 0. The DRG and SC tissue were collected during the acute phase at Days 12 and 15 (first inflammatory phase prior to de-myelination). In addition, DRG and SC tissue were collected during the chronic phase of the disease: Days 21 and 27 (during first remission), Day 36 (de-myelinating phase), and Day 45 (during the second period of remission: re-myelination phase). The DRG and SC tissue were removed to conduct molecular gene and protein analysis. Female mice were specifically chosen because females are more predisposed to be affected by MS than males [75]. The AC and EAE groups were assessed daily for neurological disability until sacrificed. NDS were determined from mean clinical scores measured from a score of 0 (no disability) to 15 (maximal disability) [7]. The total score is the sum of the following individual scores obtained for each of the 6 specified clinical domains, according to the following specifications: tail: 0 = normal, 1 = weakness or partial paralysis, 2 = limp or complete paralysis; right and left hind limbs: 0 = normal, 1 = weakness, 2 = dragging or partial paralysis, 3 = complete paralysis; right and left forelimbs: 0 = normal, 1 = weakness, 2 = unable to support weight or partial paralysis, 3 = complete paralysis; bladder: 0 = normal, 1 = incontinent. The experimental groups were sacrificed at 12, 15, 21, 27, 36 and 45 days post induction (dpi). The disease course could be divided into acute (9–18 dpi) and chronic (18–45 dpi) phase. In order to ensure consistency in regard to the degree of the MOG-induced EAE, only animals that progressed to the NDS of 4 ± 1 for the acute disease phase and 6 ± 2 for the chronic phase were included in our study. All other EAE-induced animals that did not meet the criteria were excluded from the data analysis.

All animal experiments and procedures in this study were conducted according to protocols approved by the University of Manitoba Animal Protocol Management and Review Committee, are in full compliance with the Canadian Council on Animal Care, and are in accordance with standards set forth in the 8th Edition of Guide for the Care and Use of Laboratory Animals (Protocol Reference Number: 11-067/1/2/3 (AC 10636)).

### 4.2. Gene/Protein Assay

SC and DRG tissue were harvested at pre-determined time points for gene and protein expression analysis of MeCP2E1, MeCP2E2 and BDNF. Freshly harvested DRG and SC tissue were placed in RNA later stabilization solution (Ambion Cat#AM7020, Burlington, ON, Canada) until being processed. Whole DNA/RNA and protein was purified using commercially available kits (AllPrep DNA/RNA/protein, Qiagen, Toronto, ON, Canada) as described in our previous publications [6,8,51]. 

### 4.3. Quantitative Real Time Reverse Transcription Polymerase Chain Reaction (qRT-PCR)

RNA purified from SC tissue, converted into cDNA using the iScript™ cDNA Synthesis Kit #170-8891, Bio-Rad and Bio Rad S1000 Thermal cycler instrument (Hercules, CA, USA). Final concentration of cDNA used in qRT-PCR was 5 ng/μL. The PCR reaction was performed by the CFX96 real-time PCR detection system using SsoFast™ EvaGreen^®^ Supermix (#172-5202) following manufacturers protocols (Bio-Rad, Hercules, CA, USA). MeCP2E1 primers were forward: 5′-GGAGAGAGGGCTGTGGTAAA-3′; reverse: 5′-CTGGAGATCCTGGTCTTCTGA-3′ at annealing temperature of 56 °C. MeCP2E2 primers were forward: 5′-GGAGGAGAGACTGCTCCATAAA-3′; reverse: 5′-GGAGATCCTGGTCTTCTGACTT-3′ at annealing temperature of 59 °C. BDNF primers were forward: 5′-AGCTGAGCGTGTGTGACAGTATTAG-3′; reverse: 5′-GGGATTACACTTGGTCTCGTAGAAA-3′ at annealing temperature of 56 °C. The Δ*C*t method was applied to determine differences in gene expression levels after normalization to the arithmetic mean of glyceraldehyde 3-phosphate dehydrogenase (GAPDH) as an internal standard. 

### 4.4. Western Blot (WB) Analysis

For our NC group, we conducted WB analysis for MeCP2E1 and MeCP2E2 isoforms. DRG and SC tissues were homogenized and total supernatant protein concentration was determined for each sample using Bicinchoninic Acid (BCA) protein assay kit (Novagen, CN: 71285-3, Etobicoke, ON, Canada). For each sample, 60 µg total protein was separated by 10% Tris-Glycine gradient SDS-PAGE (Thermoscientific, CN: 0025269, Burlington, ON, Canada) at 120 V for 1 h and electrophoretically blotted onto a PVDF membrane (Immobilon, CN: IPFL00010, Etobicoke, ON, Canada) for 1 h at 0.35 A. The membranes were blocked in skim milk in TBS-T and then treated with primary antibody including: Anti-MeCP2 Isoform B polyclonal anti rabbit IgG (Cat No. ABE 333 Millipore, Etobicoke, ON, Canada) 1/500, Anti-MeCP2 E2 chicken poly clonal antibody custom-made by Dr. Rastegar (1/100), or rabbit polyclonal IgG against GAPDH (1:1000, Santa Cruz, CN: sc-25778, Mississauga, ON, Canada) overnight at 4 °C. GAPDH was utilized as a loading control to ensure equivalent amounts of protein were loaded. The primary anti-MeCP2E1 and MeCP2E2 antibodies were intended to detect the 75 kDa isoform, while the primary anti-GAPDH antibody was intended to detect 37 kDa GAPDH. After incubation with primary antibodies, membranes were washed with TBS-T and incubated with secondary antibodies. The secondary antibodies used were peroxidase-conjugated donkey anti-rabbit IgG (1:10,000, Millipore, CN: AP182P) against the anti-MeCP2E1 and GAPDH antibody and peroxidase-conjugated donkey anti-mouse IgY (1:2000, Thermoscientific, SA1-72004, Burlington, ON, Canada) against the anti-MeCP2 E2 antibody. The antigen-antibody complexes were detected using ECL detection reagent (Pierce, CN: PI32209, Burlington, ON, Canada). Membranes were exposed to chemiluminescence. However, for MeCP2E1 and E2 different exposure time were applied because of difference of efficiency between these two antibodies. Densitometry was performed using a FluorChem 8900 scanner (Alpha Innotech, Santa Clara, CA, USA) with Alpha Ease FC software. Densitometry analysis was conducted with ImageJ (verison 1.48). The individual MeCP2E1, MeCP2E2 and GAPDH band densities for each sample were normalized to an internal standard. The sample and loading control density ratios obtained relative to the internal standard were subsequently used to calculate the relative density ratio of MeCP2E1 and MeCP2E2 respectively relative to GAPDH. 

### 4.5. Quantitative Enzyme-Linked Immunosorbent Assay (ELISA) Analysis

Total protein was extracted and protein concentration was determined as described above by BCA assay [10]. Protein concentration was adjusted to 30 µg in 100 µL total sample volume. Sandwich-format ELISA was performed using a BDNF Emax ImmunoAssay System (Promega, Madison, WI, USA, cat# G7611) according to manufacturer’s instructions. BDNF protein concentration was interpolated from a standard curve with a range of 7.8–500 pg/mL. 

### 4.6. Statistical Analysis

Statistics was performed using GraphPad Prism version 5.04 for Windows, GraphPad Software, San Diego, CA, USA. Data from all samples analyzed from each animal group are reported as mean ± standard error of the mean (SEM). Statistical analysis for ELISA, WB and Real Time-PCR (RT-PCR) was performed using two-way analysis of variance (ANOVA) followed by a Bonferroni post hoc test. 

## 5. Conclusions

The focus of this manuscript was to present the molecular changes in the biological targets that were involved in causing neurological disability through the molecular mechanisms that facilitate myelin damage. However, we also realize that the EAE-induced changes identified for MeCP2 and BDNF may be a cause rather than a consequence of the associated myelin damage following an immune system attack on CNS myelin. Although we have provided data that suggest MeCP2E1 is involved in the transcriptional repression of BDNF, further studies involving MeCP2E1 knockouts must be conducted to confirm its regulation of BDNF in an animal model of MS. Furthermore, our results suggest the potential importance of tracking the expression ratios between MeCP2E1 and MeCP2E2 isoforms. Henceforth, a potential therapeutic implication of this research would be to design a screening test in those patients pre-disposed to the known risk factors for MS and subject them to biological testing of the MeCPE1/MeCP2E2 ratio. For example, patients presenting with a high ratio may be at greater risk for going on to develop MS and therefore, could serve as a useful diagnostic screening test that could be added to the battery of diagnostic tests currently used to diagnose MS patients [76,77]. Thus, MeCP2 represents a key biological target molecule where epigenetic interventional strategies aimed at both DNA methylation and histone modifications could be optimized to generate a novel therapeutic drug to halt myelin damage associated with MS [78]. Therefore, studying the link between MeCP2 and BDNF and other downstream biological targets such as NGF [5,10], TNFα [6], and CX3CL1 [9] in MS may lead to new epigenetic therapeutic approaches to promote re-myelination and myelin repair in MS.
